# Supplementary material for: Cell-Type-Specific Complement Profiling in the ABCA4−/− Mouse Model of Stargardt Disease
Source: Int J Mol Sci. 2020 Nov 11;21(22):8468. doi: 10.3390/ijms21228468 (PMC7697683; doi:10.3390/ijms21228468)
Supplement: Supplementary file 1 [file ijms-21-08468-s001.pdf]

Yassin Jabri [1,†], Josef Biber [2,†], Nundehui Diaz-Lezama [3], Antje Grosche [4,*,‡] and Diana Pauly [5,*,‡]

1 Department of Experimental Ophthalmology, Eye Clinic, University Hospital Regensburg, Regensburg, Germany, yassin.jabri@ukr.de
2 Department of Physiological Genomics, Biomedical Center, Ludwig Maximilians University Munich, Planegg-Martinsried, Germany, Josef.biber@bmc.med.lmu.de
3 Department of Physiological Genomics, Biomedical Center, Ludwig Maximilians University Munich, Planegg-Martinsried, Germany, Nundehui.Diaz-Lezama@bmc.med.lmu.de

Supplementary Data

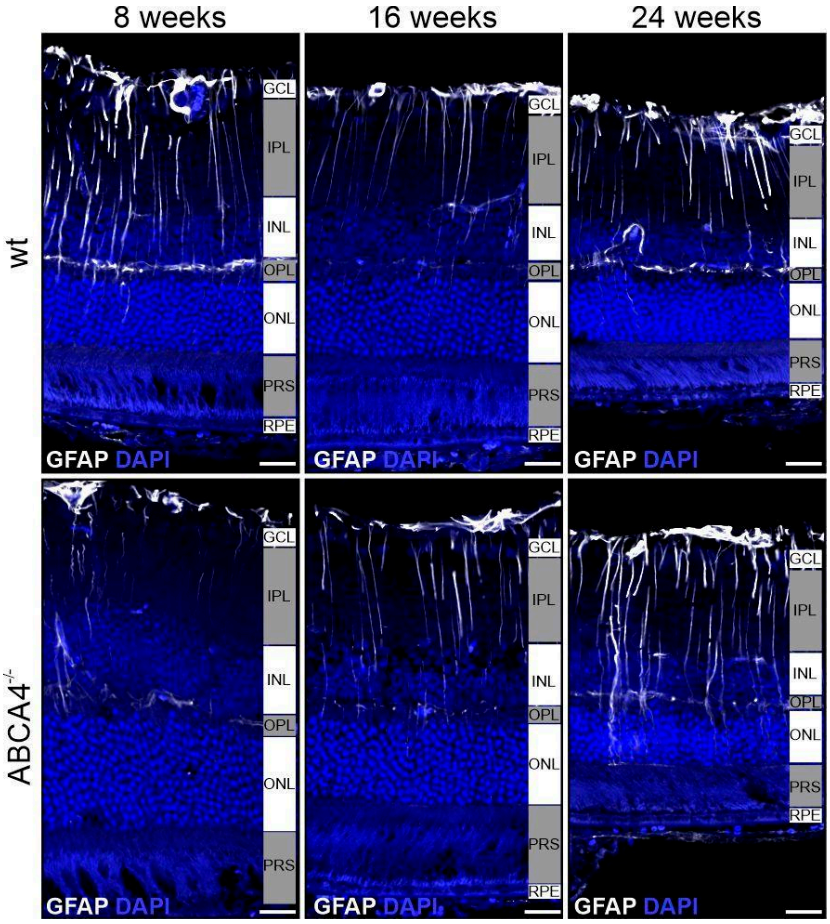

Figure S1. Retinal degeneration and concomitant Müller cell gliosis in wild type and ABCA4$^{-/-}$ mice.

In line with our cell quantification data (Figure. S1) and presumably because of the albino background, consecutive degeneration especially of photoreceptors in both genotypes was observed with aging that was accompanied with moderate Müller cell gliosis as indicated by the expression of glial fibrillary acidic protein (GFAP) already in young animals 8 weeks of age. GCL, ganglion cell layer; IPL, inner plexiform layer; INL, inner nuclear layer; OPL, outer plexiform layer, ONL, outer nuclear layer; PRS, photoreceptor segments. Scale bars, 20 µm.

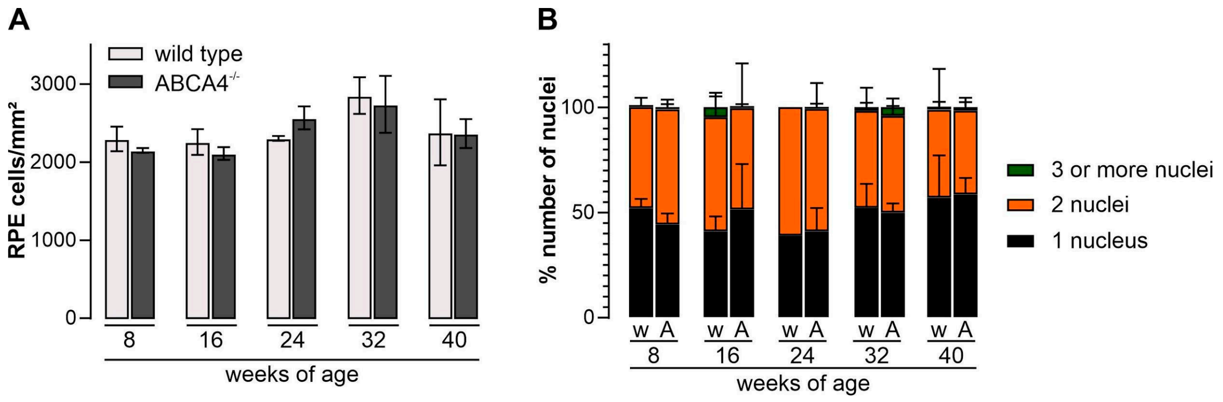

Figure S2. Characterization of the RPE integrity in ABCA4$^{-/-}$ mice.

A The total RPE cell number in both genotypes was quantified in eye cup flatmount preparations. No significant difference between genotypes was observed at any age.

B The ratio of RPE cells carrying a single nucleus, two nuclei or even more was stable with aging and did not differ between both genotypes.

A, B Bars represent mean values ± SEM from 2-5 animals per group.

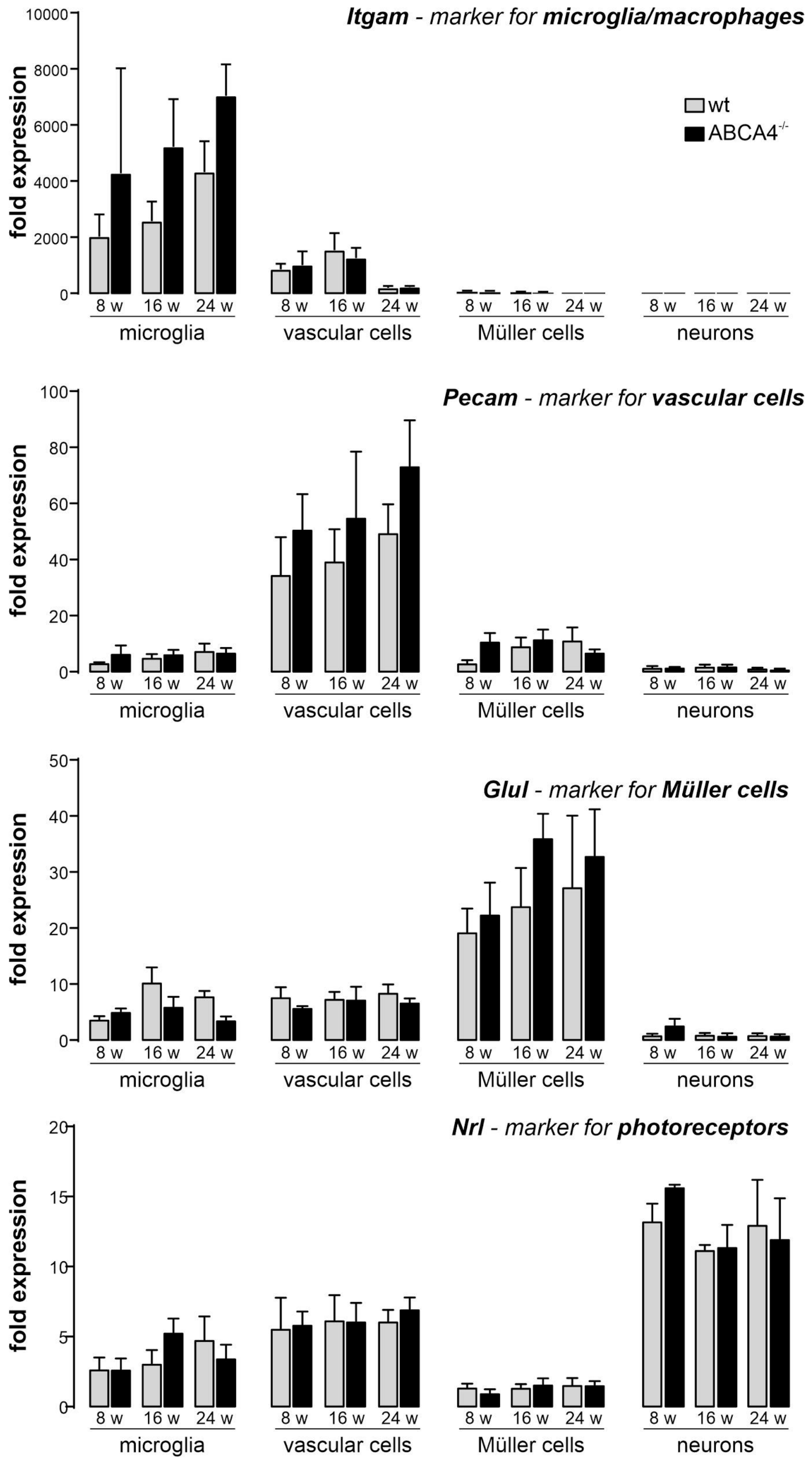

Figure S3. Immunomagnetic separation results in successful enrichment of microglia, vascular cells, Müller cells and retinal neurons irrespective of age and genotype of the mice. Retinal cell populations were enriched by immunomagnetic cell separation and characterized by qRT-PCR detecting the expression levels of specific marker genes: Microglia (and putatively co-enriched macrophages)

specifically express *itgam* (also known as *cd11b*). Vascular cell enrichment was proven by strong expression of *pecam*. *Glul* is a marker for the Müller cell fraction. Retinal neurons were characterized by an enhanced detection of the photoreceptor-specific *nrl* mRNA compared to the other cell populations. Expression of marker genes was normalized to that of *idh3b*, the housekeeping gene used throughout this study. Bars represent mean values $\pm$ SEM for cell preparations from mice 8, 16 and 24 weeks of age (n= 4 – 6 for each age). W, weeks of age; wt, wild type.

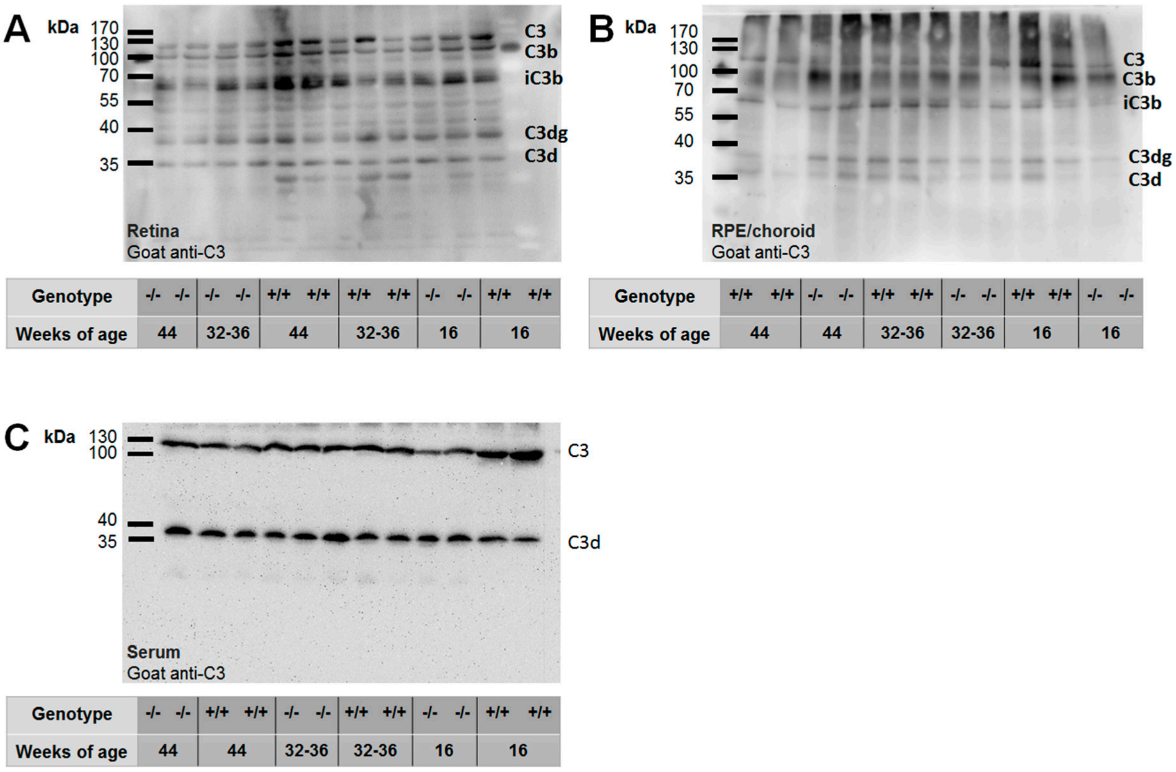

Figure S4. Western blot analysis and immunostaining of complement factor C3 in the RPE/Choroid, retina and serum of aging albino wild type and ABCA4$^{-/-}$ mice. (C3d His1002-Arg1303 (Accession # P01027) specific)

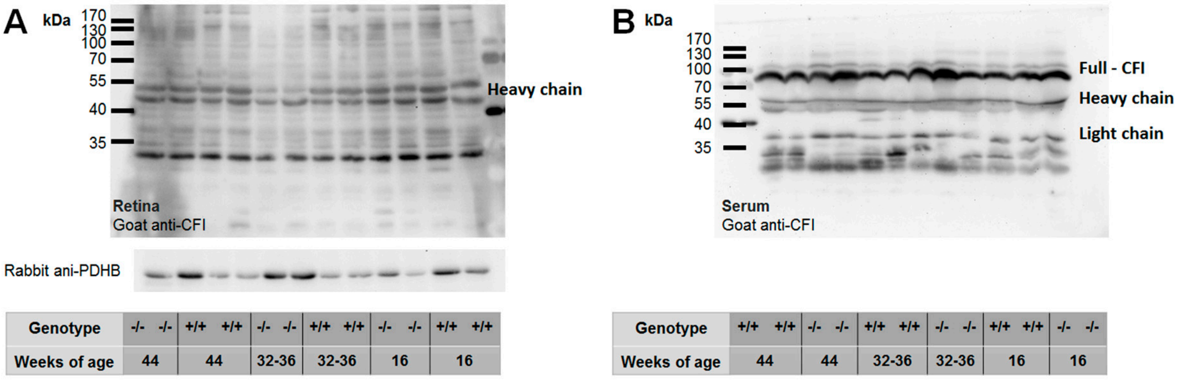

Figure S5. Detection of complement factor I in the retina and serum of aging albino wild type and ABCA4$^{-/-}$ mice.

Supplementary Table 1

Age in weeks (w)	8 w	16 w	24 w	32 w	40-44 w	
Histology						RETINA
DAPI+ GCL	n.d.	0.500	**0.014**	0.050	**0.008**	
DAPI+ INL	n.d.	0.200	0.240	0.100	0.450	
DAPI+ ONL	n.d.	0.500	0.420	0.200	0.500	
microglia INR number	n.d.	n.d.	0.413	**0.024**	**0.024**	
microglia OR	n.d.	n.d.	0.800	0.869	0.869	
Complement expression						
cfi transcript - microglia	0.100	0.691	**0.046**	n.d.	n.d.	
C3b/C3 protein ratio (whole retina)	n.d.	**p<0.05**	n.d.	p>0.05	p>0.05	
C3b/C3i protein ratio (whole retina)	n.d.	p>0.05	n.d.	p>0.05	p>0.05	
C3d/C3 protein ratio (whole retina)	n.d.	**p<0.05**	n.d.	p>0.05	p>0.05	
CFI heavy chain	n.d.	p>0.05	n.d.	p>0.05	**p<0.05**	
Histology						RPE
RPE cell number	0.200	0.400	0.333	0.500	0.999	
RPE autofluorescence	0.400	0.050	**0.008**	**0.008**	**0.004**	
Complement expression						
c3 RPE	0.400	0.999	**0.260**	n.d.	n.d.	
C3b/C3	n.d.	p>0.05	n.d.	p>0.05	**p<0.05**	
C3b/C3i	n.d.	p>0.05	n.d.	p>0.05	**p<0.05**	
C3d/C3	n.d.	p>0.05	n.d.	p>0.05	p>0.05	
Complement expression						Serum
C3d/C3	n.d.	**p<0.05**	n.d.	p>0.05	p>0.05	
CFI heavy chain	n.d.	p>0.05	n.d.	p>0.05	p>0.05	
